# Depressive symptoms following natural disaster in Korea: psychometric properties of the Center for Epidemiologic Studies Depression Scale

**DOI:** 10.1186/s12955-017-0811-9

**Published:** 2017-11-28

**Authors:** Sungkun Cho, Yongrae Cho

**Affiliations:** 1 Department of Psychology, Chungnam National University, 99 Daehak-ro, Yuseong-gu, Daejeon, 34134 Republic of Korea; 2 Department of Psychology, Hallym University, 1 Hallymdaehak-gil, Chuncheon, Gangwon-do 24252 Republic of Korea

**Keywords:** Natural disaster, Depressive symptoms, Center for Epidemiologic Studies Depression Scale, Psychometric properties, Korean

## Abstract

**Background:**

Depressive symptoms have been recognized as one of the most frequent complaints among natural disaster survivors. One of the most frequently used self-report measures of depressive symptoms is the Center for Epidemiologic Studies Depression Scale (CES-D). To our knowledge, no study has yet examined the factor structure, reliability, and validity of the CES-D in a sample of natural disaster survivors. Thus, the present study investigated the factor structure, reliability, and validity of a Korean language version of the CES-D (KCES-D) for natural disaster survivors.

**Methods:**

We utilized two archived datasets collected independently for two different periods in 2008 in the same region of Korea (*n* = 192 for sample 1; *n* = 148 for sample 2). Participants were survivors of torrential rains in the mid-eastern region of the Korean peninsula. For analysis, Samples 1 and 2 were merged (*N* = 340). Confirmatory factor analysis was performed to evaluate the one-factor model, the four-factor model, and the bi-factor models, as well as the second-order factor model. Composite reliability was computed to examine the internal consistency of the KCES-D total and subscale scores. Finally, Pearson’s *r* was computed to examine the relationship between the KCES-D and the trauma-related measures.

**Results:**

The four-factor model provided the best fit to the data among the alternatives. The KCES-D showed adequate internal consistency, except for the ‘interpersonal difficulties’ subscale. Also regarding concurrent validity, weak to moderate positive correlations were observed between the KCES-D and the trauma-related measures.

**Conclusions:**

The results support the four-factor model and indicate that the KCES-D has adequate psychometric properties for natural disaster survivors. If these findings are further confirmed, the KCES-D can be used as a useful, rapid, and inexpensive screening tool for assessing depressive symptoms in natural disaster survivors.

## Background

Depressive symptoms have been recognized as one of the most frequent complaints among natural disaster survivors [1, 2]. It has been reported that depressive symptoms and depressive disorders of trauma survivors are associated with their poor health-related quality of life [3–5]. The development, validation, and utilization of instruments to measure depressive symptoms in trauma survivors are essential for planning and applying interventions designed to reduce depressive symptoms and improve quality of life. A number of self-report measures have been developed to evaluate the degree of depressive symptoms. One of the most frequently used self-report measures of depressive symptoms is the Center for Epidemiologic Studies Depression Scale (CES-D).

The CES-D contains 20 items rated on a 4-point Likert scale, ranging from 0 to 3 (total range 0-60) [6]. There is an extensive body of research demonstrating its sound psychometric properties, including high internal consistency, fair stability, good concurrent, convergent, and discriminant validity in a variety of samples (e.g., [7–10]). Numerous investigations have been conducted to investigate the factor structure of the CES-D. The original study found a four-factor structure in the general population; ‘depressive affect,’ ‘positive affect,’ ‘somatic and retarded activity,’ and ‘interpersonal difficulties’ [6]. The four original factors have been extensively replicated (e.g., [10–13]) and confirmed with meta-analytic methods [14]. However, many other studies have suggested that the factor structure of the CES-D may vary with the study populations and culture. For example, several studies have suggested the presence of one-, two-, three-, four-, five-, and seven-factor structures in different populations and subpopulations (e.g., [11, 15]). To our knowledge, no study has yet examined the factor structure, reliability, and validity of the CES-D in a sample of natural disaster survivors. Although the CES-D has shown adequate psychometric properties in Korean clinical and nonclinical samples [8, 16], it has not been validated in a sample of Korean natural disaster survivors.

Thus, the objectives of the present study were twofold. First, we aimed to identify the best-fitting factor model for a Korean language version of the CES-D (KCES-D) in a sample of natural disaster survivors. To this end, confirmatory factor analyses (CFAs) were performed to evaluate the one-factor model, the four-factor model, and the bi-factor models (one general factor and four specific factors or three specific factors excluding depressive affect factor) [17], as well as the second-order model (the four-factor model with a single second-order factor) [10]. Second, we attempted to investigate the reliability and validity of the KCES-D for natural disaster survivors. As evidence for concurrent validity, we hypothesized that the KCES-D has a moderately positive correlation with the Impact of Event Scale-Revised (IES-R) [18], Peri-Traumatic Dissociation (PTD) Scale [19], Post-Traumatic Cognition (PTC) Scale [20], and Post-Traumatic Social Support (PTSS) Scale [21].

## Methods

### Participants

We utilized two archived datasets collected independently for two different periods in 2008 in the same region of Korea (*n* = 192, 60.4% women for sample 1; *n* = 148, 50.0% women for sample 2). Participants were survivors of torrential rains in the mid-eastern region of the Korean peninsula. Data from one participant who did not respond to half of the KCES-D was excluded, resulting in a final sample size of 340. Their sociodemographic characteristics are presented in Table 1. Data were analyzed with informed consent by participants.

**Table 1:** Sociodemographic characteristics of the samples

Variable	Sample 1 (*n* = 192)	Sample 2 (*n* = 148)
Age (%)		
20 year-old or under	0.0	2.2
20-29 year-old	6.8	1.5
30-39 year-old	9.4	5.8
40-49 year-old	13.6	12.4
50-59 year-old	24.6	26.3
60-69 year-old	17.8	24.1
70 year-old or over	27.7	25.5
Sex (%)		
Men	39.6	50.0
Women	60.4	50.0
Marital status (%)		
Married	74.0	74.1
Non-married	26.0	25.9
Education level (%)		
≥ Middle school	48.0	35.2

### Measures

Depressive symptoms were assessed using the CES-D [6]. Four variables were used to assess the concurrent validity of the KCES-D. Post-traumatic stress symptoms were assessed by the IES-R [18], peri-traumatic dissociation was assessed by the PTD Scale [19], post-traumatic cognition by the PTC Scale (PTCS) [20], and social support by the PTSS Scale [21]. Korean versions of these measures showed good reliability and validity [8, 19–21].

### Statistical analysis

All analyses were performed using the SPSS 22.0 and Mplus 7.4 software for Windows. A sample size of at least 10 cases for each item is recommended for confirmatory factor analysis [22], and thus Samples 1 and 2 were merged (*N* = 340). We estimated the factor structure using weighted least squares with mean- and variance-adjusted (WLSMV). Radloff suggested only the use of CES-D total score [6], and the correlated four-factor (i.e., first-order) model of Radloff [6] was compared to the one-factor model, the bi-factor models [17], and the second-order factor model. Goodness-of-fit of the model was assessed using the root-mean square error of approximation (RMSEA), comparative fit index (CFI), and Tucker-Lewis index (TLI). For the RMSEA, values < .05 indicate a close fit to the data, values in the range of .05 to .08 indicate a fair fit, and that values greater than .10 indicate a poor fit [23]. For the CFI and TLI, values > .90 indicate a good fit to the data [24]. Composite reliability was computed to examine the internal consistency of the total and subscale scores of the KCES-D. Although Cronbach’s alpha is the most commonly used measure for the reliability of the scales, it presupposes a tau-equivalent measurement model; otherwise it could be biased. The measurement models of the scales are clearly congeneric. Therefore, we used composite reliability as an appropriate measure for reliability in this study [25, 26]. Finally, Pearson’s *r* was computed to examine the relationships between the KCES-D and the trauma-related measures.

## Results

**Fig. 1:**
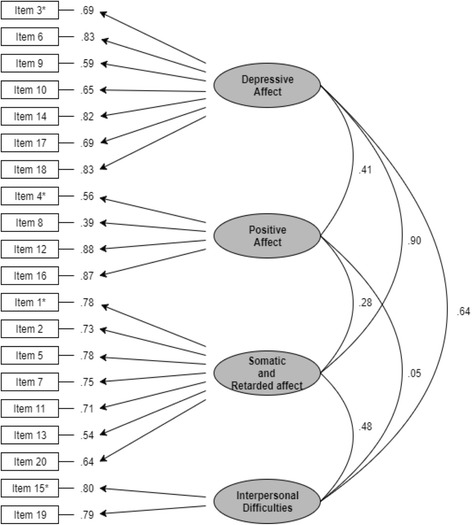
The Path diagram of the correlated four-factor model of the KCES-D (standardized solution). Asterisks(*) denote Items fixed at 1.00 for model identification purposes and scaling

Confirmatory factor analysis indicated a poor fit for the one-factor model and a good fit for the correlated four-factor (i.e., first-order) model (see Table 2). Improper solutions [model specification errors such as a non-positive definite latent variable covariance matrix (psi)] were obtained for both the bi-factor models and the second-order factor model. Figure 1 display the path diagram of the correlated four-factor model, and Table 3 presents descriptive statistics for the KCES-D. The internal consistency for ‘depressive affect,’ ‘positive affect,’ ‘somatic and retarded activity,’ ‘interpersonal difficulties,’ and the total scale of the KCES-D yielded composite reliability = .84, .74, .83, .63, and .94, respectively. The distribution of the total scores of the KCES-D indicated that 45.4% of the sample had scores of 21 or higher (cutoff score) [27]. Table 4 presents the Pearson correlations between the KCES-D and the trauma-related measures. The KCES-D had weak to moderate positive correlations with the IES-R, PTD Scale, PTC Scale, and PTSS Scale.

**Table 2:** Goodness-of-fit indices for the KCES-D (*N* = 340)

Model	χ^2^	*df*	RMSEA (90% CI)	CFI	TLI
one-factor	1004.02^***^	170	.12 (.11-.13)	.83	.81
four-factor	340.45^***^	164	.06 (.05-.07)	.96	.96

Note: RMSEA: Root-mean-square error of approximation; CFI: Comparative fit index; TLI = Tucker-Lewis index. ***p < .001

**Table 3:** Descriptive statistics for subscale and total scores of the KCES-D

Subscale	# items	Possible range	*M*	*SD*	Intercorrelations			
					1	2	3	4
DA	7	0-21	5.08	4.66				
PA	4	0-12	8.61	2.86	.26^***^			
SR	7	0-21	6.48	5.08	.75^***^	.17^**^		
ID	2	0-6	.76	1.25	.44^***^	.01	.30^***^	
Total score	20	0-60	20.77	10.67	.91^***^	.47^***^	.89^***^	.47^***^

Note: DA: depressive affect; PA: positive affect; SR: somatic and retarded activity; ID: interpersonal difficulties. **p < .01, ***p < .001

**Table 4:** Correlations between the KCES-D subscale and total scores and the PTSD-related variables

	DA	PA	SR	ID	Total score
Post-traumatic stress symptoms	.55^***^	.09	.59^***^	.31^***^	.59^***^
Peri-traumatic dissociation	.19^**^	.04	.23^***^	.16^**^	.22^***^
Post-traumatic negative cognitions	.51^***^	.13^*^	.48^***^	.33^***^	.52^***^
Post-traumatic low social support	.35^***^	.14^**^	.23^***^	.24^***^	.33^***^

Note: DA: depressive affect; PA: positive affect; SR: somatic and retarded activity; ID: interpersonal difficulties. *p < .05, ** *p* < .01, ***p < .001

## Discussion

To the best of our knowledge, this study is the first to investigate the psychometric properties of the KCES-D for natural disaster survivors. The KCES-D showed a good fit for the correlated four-factor structure and adequate internal consistency, indicating a high interrelatedness of items, except for the ‘interpersonal difficulties’ subscale (composite reliability = .63), possibly due to its small number of items [28]. In addition, 45.4% of the current sample had scores of 21 or higher (cutoff score), which is somewhat higher than the rate (25.0%) found in a community sample of Korean adults [27]. Regarding concurrent validity, weak to moderate positive correlations were observed between the KCES-D and the trauma-related measures. This finding suggests that greater depressive symptoms are associated with greater post-traumatic stress symptoms, peri-traumatic dissociation, and post-traumatic cognition, as well as lower social support. Overall, the KCES-D appears to be an adequate instrument for assessing depressive symptoms in natural disaster survivors.

The four-factor structure is consistent with that in the Korean study [8] as well as the original study [6]. Notably, the present study findings indicated a relatively high mean score (positively-keyed items for which a higher score indicates a lower positive affect) on the positive affect subscale. However, this is not surprising given the similar findings consistently shown in East Asia (e.g., Korea, Japan, China, Philippines) [29]. In East Asia, people tend to suppress the expression of positive emotion, especially in the presence of others and are less likely to endorse positive affect, compared to those in North and South America [30, 31]. Given this, the cutoff score (21 or higher) of the KCES-D has been established higher than the CES-D (16 or higher) [27].

Radloff suggested only the use of CES-D total score [6], but the study findings did not seem to provide such justification. For example, although its internal consistency coefficient for the total score was high (composite reliability = .94) and exceeded that for the subscale scores, the one-factor model of the KCES-D did not fit the data well. Further, improper solutions were obtained for both the bi-factor models and the second-order factor model, and the four subscales of the KCES-D showed weak to strong intercorrelations and varied correlations with each trauma-related measure. Thus, its total score needs to be used carefully and further research is needed to examine whether the subscale scores have unique impacts on various trauma-related measures using multiple regression analysis [32].

Interestingly, there was a very low correlation between the positive affect subscale and the interpersonal difficulties subscale of the KCES-D. Also, the positive affect subscale had a weak correlation (*r* = .04 - .14) with the trauma-related measures. Quality of life includes positive and negative affect [33], and generally the lower the positive affect and the higher the negative affect represents lower quality of life [34]. However, the results do not seem to support this, and positive affect appeared to be mutually independent of quality of life, unlike negative affect. There are two possible explanations for these results. First, the tripartite model of anxiety and depression [35] consists of symptoms in three groups: 1) ‘general distress’ which includes the common elements of both anxiety and depression, 2) ‘physiological hyperarousal’ that is exclusive to anxiety, and 3) ‘anhedonia’ that is exclusive to depression. Given that the positive affect subscale may be exclusive to depression, it is possible to have such low correlation. Second, the other three subscales of the KCES-D and the trauma-related scales consist of negatively-keyed items (straightforward scoring), whereas the positive affect subscale consists of positively-keyed items (reverse scoring). Thus, it is possible that the different scoring systems elicited different responses from participants [36, 37].

This study has some limitations. First, there was no diagnostic assessment for either depression or post-traumatic stress disorder. Second, this study employed natural disaster survivors living in rural areas and thus should be interpreted with caution in generalizing the study findings. Third, the test-retest stability of the KCES-D was not assessed. Fourth, improper solutions were obtained for both the bi-factor models and the second-order factor model, which may indicate wrong model specifications or low number of participants. Thus, further research is needed to assess the models with higher number of participants.

## Conclusions

Nevertheless, the results support the correlated four-factor model and indicate that the KCES-D has adequate psychometric properties concerning application for natural disaster survivors. If these findings are further confirmed, the KCES-D can be used as a useful, rapid, and inexpensive screening tool for assessing depressive symptoms in natural disaster survivors. Certainly, the KCES-D alone should not be used as a diagnosis and treatment recommendation for depression in natural disaster survivors [38].

## Abbreviations

CES-D: Center for Epidemiologic Studies Depression Scale
CFI: Comparative Fit Index
IES-R: Impact of Event Scale-Revised
KCES-D: Korean version of the Center for Epidemiologic Studies Depression Scale
PTC: Post-Traumatic Cognition
PTD: Peri-Traumatic Dissociation
PTSS: Post-Traumatic Social Support
RMSEA: Root-Mean Square Error of Approximation
TLI: Tucker-Lewis index
